# Predictors of Change in Wellbeing and Mental Health of Parents of Autistic Pre-Schoolers

**DOI:** 10.1007/s10803-024-06471-7

**Published:** 2024-07-26

**Authors:** Cherie C. Green, Jodie Smith, Catherine A. Bent, Lacey Chetcuti, Mirko Uljarević, Paul R. Benson, Kristelle Hudry

**Affiliations:** 1 Department of Psychology, Counselling and Therapy, School of Psychology and Public Health, La Trobe University, Melbourne, Australia; 2 School of Allied Health, Human Services and Sport, La Trobe University, Melbourne, Australia; 3 Melbourne School of Psychological Sciences, Faculty of Medicine, Dentistry and Health Sciences, The University of Melbourne, Melbourne, Australia; 4 Stanford Autism Centre, Department of Psychiatry and Behavioural Sciences, Child and Adolescent Psychiatry, School of Medicine, Stanford University, Stanford, CA USA; 5 Department of Sociology, University of Massachusetts, Boston, USA; 6 Telethon Kids Institute, WA Nedlands, Australia

**Keywords:** Parents, Pre-school children, Mental health, Wellbeing, Longitudinal, Change

## Abstract

Parenting is a rewarding experience but is not without its challenges. Parents of Autistic children face additional challenges, and as a result can experience lower levels of wellbeing and more mental health problems (i.e., depression, anxiety, stress). Previous studies have identified concurrent correlates of wellbeing and mental health. However, few have investigated predictors of subsequent wellbeing and mental health, or of change over time, among parents of pre-school aged autistic children. We examined child-, parent-, and family/sociodemographic factors associated with change in parents’ mental health and wellbeing across three timepoints (spanning approximately one year) among 53 parents of Autistic pre-schoolers (*M* = 35.48, *SD* = 6.36 months. At each timepoint, parents reported lower wellbeing and greater mental health difficulties compared to normative data. There was no significant group-level change over time in parent outcomes. However, individual variability in short-term (~ 5 months) wellbeing and mental health change was predicted by a combination of child- and parent-related factors, while variability in medium-term (~ 10 months) change was predicted by parent factors alone. Parents’ description of their child and their relationship predicted change in both wellbeing and mental health. Furthermore, participating in a parent-mediated intervention (available to a subgroup) was a significant predictor of change in wellbeing. Our findings highlight potentially modifiable factors (e.g., learning healthier coping strategies) that may positively impact both short- and medium-term change in parental outcomes.

**Supplementary Information:**

The online version contains supplementary material available at 10.1007/s10803-024-06471-7.

## Introduction

While raising an Autistic child can be rewarding (Potter, 2016), many parents experience more mental health difficulties (i.e., chronic stress, anxiety, and depression) and lower wellbeing (i.e., life satisfaction, autonomy, purpose, connectedness to people, balance of positive and negative emotions) than parents of non-Autistic children (Green et al., 2021; Salomone et al., 2018; Schnabel et al., 2020). This may be particularly evident during early childhood, where parents have reported increasing levels of parenting-related stress following their child’s diagnosis (Green, 2024). In an adaptation of Belsky’s (1984) model of parenting, it has been posited that a combination of parent characteristics, child characteristics, and family social environment impact parenting in early childhood (Taraban & Shaw, 2018). Among families of young Autistic children, additional parenting responsibilities and challenges further add to complexity of parenting in early childhood. In particular, stigma (den Houting et al., 2021; Smith et al., 2023) and inadequate access to quality resources (Smith et al., 2023) may further contribute to parenting stress, in line with minority stress theory (Meyer, 2003), which takes into account the added stressors experienced by individuals from minority groups and communities, including the Autism community (Gunty, 2021). This may be especially true in Australia; despite an increasing need for supports following diagnosis, there has not been a proportionate increase in services, leaving many families struggling to cope, particularly marginalised families (Australian Government, 2023; Commonwealth of Australia, 2023; Smith et al., 2023). These added stressors and unique circumstances may be at least partially contributing to mental health challenges and lower wellbeing in parents of young Autistic children.

Individual differences in mental health and wellbeing in parents of Autistic children have been linked to a broad range of factors. Some such factors relate to characteristics of parents, including use of coping strategies (Benson, 2014), social supports (Benson, 2020), and mindfulness (Cheung et al., 2019; Green et al., 2021), as well as their personality (Green et al., 2021) and autistic traits (Ingersoll & Hambrick, 2011; Pruitt et al., 2018). Other factors associated with parent wellbeing and mental health challenges relate to child characteristics, including autistic presentation (Green et al., 2021; Ingersoll & Hambrick, 2011; Mathew et al., 2019) and emotional and behaviour problems (Cheung et al., 2019; Pruitt et al., 2018; Salomone et al., 2018; Smith et al., 2021), as well as the parent–child relationship quality (Hastings et al., 2006). In addition, socioeconomic status and cultural background have also been shown to correlate with parental mental health and wellbeing (Mathew et al., 2019).

What remains unclear is how the aforementioned factors may be differentially associated with parental mental health versus wellbeing, and their relative predictive value. Mental health challenges and wellbeing have been presumed to represent opposite ends of the same construct (Kinderman et al., 2015). However, it is increasingly clear that mental health and wellbeing are related yet distinct constructs, with unique determinants (Cai et al., 2020; Green et al., 2021; Kinderman et al., 2015; Patalay & Fitzsimons, 2016; Salomone et al., 2018). For example, amongst parents of school-aged Autistic children, mental health challenges have been shown to be concurrently associated with measures of child factors (i.e., reduced daily living skills, cognitive impairment, and greater emotional and behavioural problems), parent factors (i.e., higher education level), and family sociodemographic factors (i.e., lower household income), whereas wellbeing was associated with child characteristics alone (i.e., fewer emotional and behavioural problems) (Salomone et al., 2018). However, among parents of Autistic pre-schoolers, a different pattern of characteristics associated with concurrent parental mental health and wellbeing has been identified (Green et al., 2021). Specifically, mental health difficulties were predicted by a combination of child- (i.e., parent-reported autism traits) and parent characteristics (i.e., trait negative emotionality), with wellbeing predicted by parent factors alone (i.e., trait extraversion/sociability, mindfulness, and mental health) (Green et al., 2021). Although the specific predictors of mental health challenges and wellbeing were different across these two studies (Green et al., 2021; Salomone et al., 2018), mental health challenges were consistently shown to be influenced by a broader range of factors compared to wellbeing.

While there is growing evidence that mental health and wellbeing have unique determinants among parents of Autistic children (Cai et al., 2020; Green et al., 2021; Salomone et al., 2018), the cross-sectional design of existing work limits the inferences that can be drawn, and longitudinal studies have to date largely focused on parents of Autistic school-aged and adolescent children. Less work is dedicated to parents of younger, more recently-diagnosed children who may be particularly vulnerable to mental health and wellbeing impacts (Hickey et al., 2021). Parent-mediated interventions that focus on child outcomes have been suggested as indirect avenue to support parental mental health and wellbeing during early childhood (Estes et al., 2019). However, the evidence for parental benefits from these interventions is less substantial than for children (see Green, 2024; Leadbitter et al., 2018; Oono et al., 2013). For example, Green (2024) recently found that Autistic pre-schoolers made substantial developmental gains regardless of the type of support they received, and that additional parent-mediated intervention did not improve child or parent outcomes. However, it was suggested that using alternative methods of analysis, such as the reliable change index (RCI; Jacobson & Truax, 1992), may be a psychometrically reliable way of measuring individual-level change, compared to other methods used to assess group mean-level change over time.

### The Current Study

Operating within the framework of Taraban and Shaw’s (2018) model of parenting, we examined which, among a range of child, parent, and family/socioeconomic factors, predicted change over time in self-reported mental health and wellbeing in a sample of parents of Autistic pre-schoolers, accessing community group/centre-based and/or parent-mediated supports. Based on past work, we hypothesised that baseline parent measures (i.e., mindfulness, coping strategies, and social supports), child features (i.e., autistic presentation, emotional and behavioural problems), parent–child relationship quality, and family/socioeconomic factors (i.e., income, cultural background) would predict change (i.e., RCI z-scores) in both parental mental health and wellbeing. We were particularly interested to see whether there would be common or unique predictors of change in parental mental health challenges and wellbeing, making no particular predictions given the limited amount of comparative research within this age range. As parent-mediated supports have been suggested to benefit parent mental health and wellbeing (Leadbitter et al., 2018; Oono et al., 2013), we further hypothesised that participation in this particular approach (vs. centre/group-based supports) might also evidence benefits.

## Method

### Participants and Procedure

Parent–child dyads (N = 53) participated in a broader evaluation of Autistic children’s outcomes following early intervention; engagement with community-based service providers (n = 17) or a university co-located early intervention service (n = 36). Study eligibility criteria have been previously reported (see Green, 2024; Smith et al., 2021, 2022). Briefly, children were aged 17–43 months with a confirmed Autism diagnosis, and no parent had significant and unmanaged depression or anxiety such that the demands of study participation might exacerbate their condition.

**Fig. 1:**
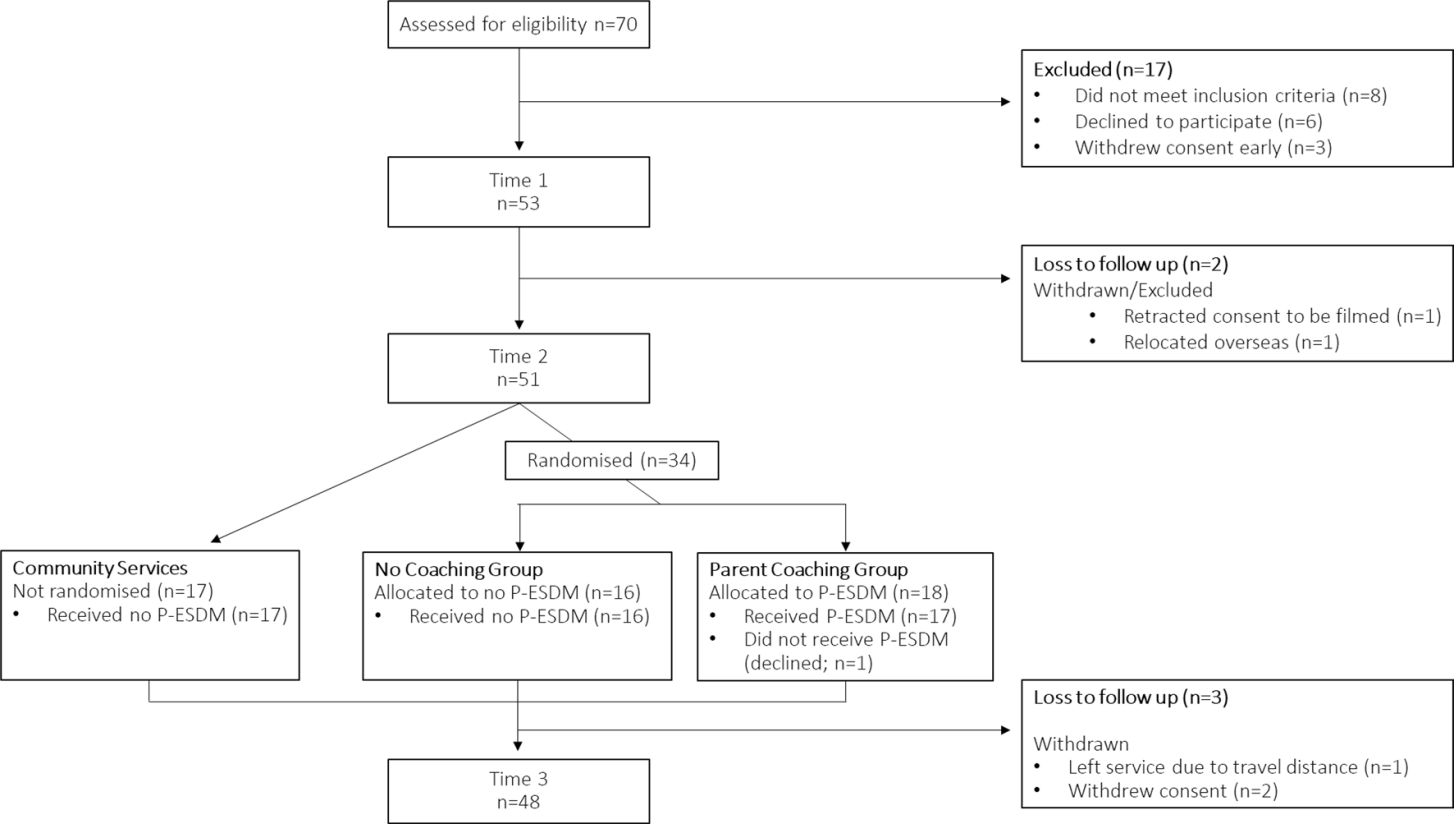
Study enrolment and participation charactersitics. Eligible participants were assessed at three timepoints. Immediately after Time 2, a subset of participants were randomised to be offered P-ESDM

The data presented here were obtained at three timepoints **(**Fig. 1**)**: study intake (Time 1; T1), and approximately 5- (T2) and 10 months (T3) later. A subset of families (n = 18), selected at random, were offered parent-mediated support—specifically parent delivered early start denver model (P-ESDM; Rogers et al., 2012)—between their T2 and T3 assessments, as an adjunct to their other group/centre-based service enrolment ESDM program (Rogers & Dawson, 2010; Vivanti et al., 2017, 2019) between T1 and T3. Ethics approval was obtained from La Trobe University (HREC # 16–136). Parents provided informed consent for their own and their child’s research participation.

### Measures

#### Parent and Family Context Characteristics

The Depression Anxiety Stress Scales (DASS-21; Lovibond & Lovibond, 1995) was completed at each timepoint to measure mental health challenges. The DASS-21 is a 21-item questionnaire, with items rated on a 4-point Likert-type scale (0 = *Did not apply to me at all*; 3 = *Applied to me very much, or most of the time*). Items are negatively worded (e.g., *I find it hard to wind down*); higher scores indicate more challenges across three subscales measuring depression, anxiety, and stress. Total scores were used in analyses (range 0–120). The DASS-21 has a more robust factor structure than the DASS, and high reliability and convergent validity with other comparable measures (Henry & Crawford, 2005).

The Warwick-Edinburgh Mental Wellbeing scale (WEMWBS; Tennant et al., 2007a, 2007b) was completed at each timepoint to measure wellbeing. The WEMWBS is a 14-item questionnaire measuring subjective and psychological components of wellbeing. Items are rated on a 5-point Likert-type scale (1 = *None of the time*; 5 = *All of the time*). Items are positively worded (e.g., “I’ve been feeling optimistic about the future); higher scores indicate greater wellbeing. Total scores were used in analyses (range 14–70). The WEMWBS has good validity and high reliability (Tennant et al., 2007a, 2007b) and is sensitive to change across populations and in diverse public health interventions and programs (Stewart-Brown et al., 2011).

The Clarke modification of the Holroyd Questionnaire on Resources and Stress (CQRS; Konstantareas et al., 1992) was used to measure parenting-related stress and resources at T1. The CQRS was designed for use with families with children with neurodevelopmental conditions. The CQRS comprises 78 items rated on a 4-point Likert-type scale (1 = *Strong agreement with statement*; 4 = *Strong disagreement with statement*). Statements are worded either positively (e.g., “Our relatives have been helpful”) or negatively (e.g., “I have too much responsibility”); higher scores reflect more parenting stress and fewer resources. Mean scores were used in analyses (range 1–4). The CQRS has good internal consistency, split-half reliability, and coefficient of stability, as well as acceptable construct and concurrent validities (Konstantareas et al., 1992).

The Brief COPE Scale (Carver, 1997) was used to measure coping strategies at T1. It comprises 28 items rated on a 4-point Likert scale (1 = *I haven’t been doing this at all*; 4 = *I’ve been doing this a lot*). Scores for two overarching coping styles (Carver et al., 1989) were computed—avoidant (e.g., self-distraction, denial, venting) and approach coping (e.g., emotional support, positive reframing, acceptance)—with higher scores reflecting more behaviours indicative of each coping style. The Brief COPE has been shown to have adequate factor structure (Carver, 1997) and has been used in parents of Autistic children (Benson, 2010, 2014).

The Autism-Specific Five-Minute Speech Sample (AFMSS; Benson et al., 2011) provided a measure of the quality of the parent–child relationship at T1, through the coding of indicators of expressed emotion (EE) from a five-minute speech recording of each parent’s description of their child and their relationship. Parental EE is comprised of six subcomponents, including four categorical codes: initial statement, warmth, relationship, and emotional over-involvement (EOI), rated as either *positive/neutral/negative* or *high/moderate/low*. The final two subcomponents are critical comments and positive comments, both scored as a frequency counts. EE subcomponent ratings were used in analyses. (for more details see Smith et al., 2021). The AFMSS has been shown to have good validity and reliability (Benson et al., 2011) and has been used in parents of pre-school aged children (Smith et al., 2021).

The Mindful Attention Awareness Scale (MAAS; Brown & Ryan, 2003) was used to measure mindfulness at T1. The MAAS consists of 15 items (e.g., “I could be experiencing some emotion and not be conscious of it until some time later.”) rated on a 6-point Likert scale (1 = *Almost always*; 6 = *Almost never*). The mean score was used for analyses, with higher scores indicating greater levels of mindfulness. The MAAS has good reliability and validity (Brown & Ryan, 2003), including in parents of Autistic children (Rayan & Ahmad, 2018).

The Autism Spectrum Quotient – 10 (AQ-10; Allison et al., 2012) provided a measure of parental autism traits at T1. The AQ-10 comprises 10 items (e.g., “I find it easy to ‘read between the lines’ when someone is talking to me.”) rated on a 4-point Likert scale (*Definitely agree* to *definitely disagree*). The total score was used in analyses, with higher scores reflecting more autism traits. The AQ-10 has high reliability and validity (Allison et al., 2012).

Sociodemographic information was collected for each family, including education level, household income, the primary language spoken at home, culturally and/or linguistically diverse (CALD) status, and number of children in the family (both Autistic/non-Autistic). Categorical sociodemographic variables (i.e., education, household income, siblings) were dichotomised for analyses. Some data collection occurred following onset of the COVID-19 worldwide pandemic, during which the local region experienced multiple, protracted periods of lockdown (i.e., stay-at-home order) which have been have been linked to poorer wellbeing and increased depression (Hedley et al., 2021). The effect of data collection before and during the pandemic was therefore also considered, by comparison of parent outcomes pre- and post-lockdown. Parent and family characteristics are presented in Table 1.

**Table 1:** Participant, Family, and Sociodemographic Characteristics

	N/M (%/SD)
**Parent**	53
Mother	45 (85%)
Father	8 (15%)
**Child Sex**	53
Male	43 (81%)
Female	10 (19%)
**Child Age (months) at Time 1**	35.48 (6.36)
**Parent’s Highest Level of Completed Education**	48
Secondary or Lower	7 (15%)
Tertiary	41 (85%)
**Household Income**	49
Low-income status/income support	17 (35%)
Not low income	32 (65%)
**Parent’s Cultural Background**	51
Australian	19 (37%)
Other	32 (63%)
**Primary Language**	51
English	36 (71%)
Other	15 (29%)
**Number of Children**	51
1 child	19 (37%)
> 1 child	32 (63%)
**Number of Children with Autism**	51
Simplex family	44 (86%)
Multiplex family	7 (14%)

#### Child Characteristics

The MSEL (Mullen, 1995) was used to assess children’s developmental/cognitive skills, and is a standardised assessment of verbal (Receptive and expressive language) and non-verbal (Fine motor and visual reception) abilities. A developmental quotient (DQ) was computed for analysis (i.e., age equivalent average/chronological age × 100), with scores at or near 100 reflecting skills near chronological age expectations. The MSEL has good internal reliability and strong test–retest and inter-scorer reliability (Mullen, 1995), as well as construct, convergent, and divergent validity (Swineford et al., 2015).

The ADOS-2 (Lord et al., 2012) was used to confirm diagnosis and assess level of autism traits, and was administered by research-reliable assessors. The modules used within the current study included the Toddler Module (for children aged 12–30 months), Module 1 (for children aged 31 months and older with no/limited speech), and Module 2 (for children using phrase speech). Calibrated Severity Scores (CSS; Esler et al., 2015; range 1–10) were calculated for analysis, with higher scores reflecting greater severity. The CSS has shown strong test re-test reliability across all modules (ICC = 0.71–0.89, *p* < 0.05) (Janvier et al., 2022).

The Social Communication Questionnaire (SCQ; Rutter et al., 2003) was used as a parent-report measure of child autism traits. The SCQ is a 40-item questionnaire yielding scores ranging from 0 to 39, with higher total scores again reflecting higher levels of autism traits. The SCQ has high sensitivity and specificity (Chandler et al., 2007; Rutter et al., 2003).

The Vineland Adaptive Behavior Scales 2nd Edition (VABS-II; Sparrow et al., 2005) was administered via parent interview to measure adaptive skills. The VABS-II measures adaptive behaviour across four domains: communication, daily living skills, socialisation, and motor skills. The Adaptive Behavior Composite (ABC) standard score (population *M* = 100; *SD* = 15) was computed for analysis, with higher scores reflecting greater adaptive behaviour abilities. The VABS-II has strong internal consistency, test–retest reliability, inter-interviewer reliability, and validity (Sparrow et al., 2005).

The preschool version of the Child Behavior Checklist (CBCL; Achenbach & Rescorla, 2000) was used as a parent-report measure of behavioural (externalising) and emotional (internalising) difficulties. The CBCL comprises 113 questions rated on a 3-point Likert scale (0 = *Absent*; 2 = *Occurs often*). Higher scores for each indicating greater difficulties. The CBCL has adequate sensitivity (0.66) and strong specificity (0.83) (Warnick et al., 2008).

#### Parent-Mediated Intervention

ESDM is a developmental-behavioural intervention that targets children’s social, cognitive, and language skills across various delivery formats (e.g., individual, group- and parent-coaching methods; Estes et al., 2014; Rogers & Dawson, 2010; Vivanti et al., 2017). For families offered P-ESDM, this comprised up to 13 1-h weekly clinic-based sessions: an initial child assessment and goal-setting session with parent and coach, and 12 sessions during which staff provided hands-on direct coaching of parents’ ESDM strategy use.

### Statistical Procedure

#### Change in Mental Health Challenges and Wellbeing

The presentation of and change in parents’ DASS-21 and WEMWBS total scores were characterised in three ways. First, group mean-level change across T1-T3 was examined using one-way repeated measures ANOVA. Second, DASS-21 and WEMWBS total scores for this sample were compared to normative levels using t-tests (including Australian-based norms for DASS-21, and UK-based norms for WEMWBS [as recommended by Taggart et al. (2016)]). Third, RCI z-scores (Jacobson & Truax, 1992) were computed as indicators of psychometrically reliable individual-level change, for each of DASS-21 and WEMWBS scores from T1-T2 and T1-T3.

#### Predictors of Change in Mental Health and Wellbeing

Candidate parent-, child-, and family-related predictors of DASS-21 and WEMWBS RCI z-scores were identified through initial correlations, t-tests, and ANOVAs (see Supplementary Tables 1 and 2). After applying the Benjamini-Hochberg (False Discovery Rate [FDR]) correction, initial significant findings were no longer present. This correction may be too stringent, and given that the effect sizes indicate small to medium effects (Ferguson, 2016), analyses proceeded without correction for multiple comparisons. Where multiple candidate predictors were initially identified, those most strongly associated with the given outcome (i.e., showed small to medium effect sizes) were retained for subsequent regression analyses, with a maximum of four potential predictors (i.e., one per 10 participant cases; (Hollestein et al., 2021)).

## Results

**Fig. 2:**
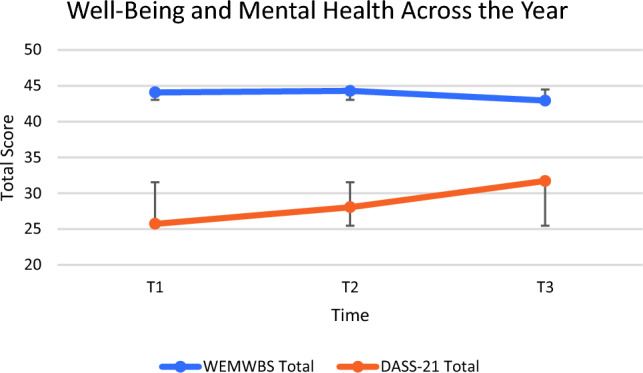
Mean Warwick-Edinburgh Mental Well-being Scale (WEMWBS) and Depression Anxiety Stress Scales (DASS-21) scores at each timepoint. Total WEMWBS scores range from 14–70 and total DASS-21 scores range from 0–120

**Fig. 3:**
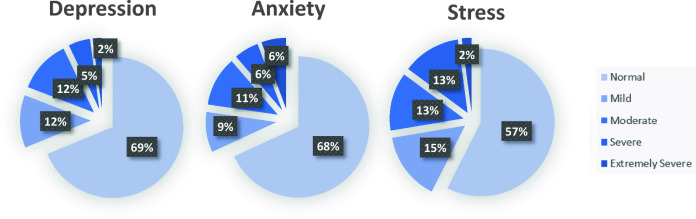
DASS-21 Subscale Ratings. Percentage of parents with Depression Anxiety Stress Scales (DASS-21) subscale scores within each of five severity ratings based on normative data

As shown in Fig. 2 there was no significant group mean-level change over time in WEMWBS [F(2.00, 76.00) = 1.112, p = 0.334, ω^2^ = 0.000], or DASS-21 total scores [F(2.00, 78.00) = 0.960, p = 0.387, ω^2^ = 0.000]. Parents in this sample consistently reported lower wellbeing and higher mental health difficulty levels than normative data **(**Table 2**)**.Approximately one-third of parents reported above ‘normal’ levels of depression, anxiety, and stress, with around 10% reporting severe or extremely severe levels (Fig. 3).

**Table 2:** Differences in Wellbeing and Mental Health Problems between the Sample and Population Norms Over Time

		Time 1			Time 2			Time 3		
		*M (SD)*	*t (df)*	*p*	*M (SD)*	*t (df)*	*p*	*M (SD)*	*t (df)*	*p*
WEMWBS Total Score										
	Population	51.607 (8.706)	−6.832 (48)	< 0.001	(Time 1)	−6.825 (47)	< 0.001	(Time 1)	−7.141 (39)	< 0.001
	Sample	44.073 (7.719)			44.292 (7.426)			42.932 (7.683)		
DASS-21 Total Score										
	Population	16.48 (19.24)	3.802 (50)	<0.001	(Time 1)	3.901 (47)	<0.001	(Time 1)	4.480 (41)	< 0.001
	Sample	25.725 (17.367)			28.042 (20.531)			31.714 (22.040)		

*WEMWBS* Warwick-Edinburgh Mental Wellbeing Scale, *DASS-21* Depression Anxiety Stress Scales

Descriptive data for parental wellbeing and mental health outcomes (T1-2 and T1-3 RCI z-scores) are presented in Table 3. While substantial individual variation was evident, the vast majority of parents were categorised as showing no reliable change over time on either measure (i.e., −1.96 < z < 1.96). Descriptive data for other T1 parent and child measures—candidate predictors of parental outcomes—are presented in Table 4.

**Table 3:** Descriptive Statistics for Parent Outcomes

	*n *(%)	*M (SD)*	Range
**Parent outcomes**			
WEMWBS reliable change index score: T1-T2	46	−0.114 (1.409)	−3.497–2.564
< −1.96: Decrease in wellbeing	4 (9%)	–	–
−1.96–1.96: No change	38 (82%)	–	–
> 1.96: Increase in wellbeing	4 (9%)	–	–
WEMWBS reliable change index score: T1-T3	39	−0.315 (1.332)	−3.963 – 2.331
< −1.96: Decrease in wellbeing	5 (13%)	–	–
−1.96–1.96: No change	33 (85%)	–	–
> 1.96: Increase in wellbeing	1 (2%)	–	–
DASS-21 Reliable change index score: T1-T2	47	0.161 (1.773)	−3.548 – 5.484
< −1.96: Increase in mental health	3 (6%)	–	–
−1.96–1.96: No change	39 (83%)	–	–
> 1.96: Decrease in mental health	5 (11%)	–	–
DASS-21 Reliable change index score: T1-T3	41	0.382 (1.942)	−4.194–6.129
< −1.96: Increase in mental health	3 (7%)	–	–
−1.96–1.96: No change	32 (78%)	–	–
> 1.96: Decrease in mental health	6 (15%)	–	–

*WEMWBS* Warwick-Edinburgh Mental Wellbeing Scale, *DASS-21* Depression Anxiety Stress Scales

**Table 4:** Descriptive Statistics for Candidate Parent and Child Predictors

	*n*	*M (SD)*	Range
**Candidate parent predictors**			
Parent age (years)	48	36.354 (4.61)	26– 48
WEMWBS wellbeing total score	49	44.073 (7.719)	25–59
DASS-21 mental health difficulties total score	51	25.725 (17.367)	0– 62
CQRS resources and stress			
Family sharing	51	2.513 (0.525)	1.380–3.570
Supports	51	2.485 (0.669)	1.00–3.750
Brief COPE coping mechanisms			
Avoidant	49	21.571 (5.05)	13– 34
Approach	49	34.857 (5.705)	23–45
MAAS mindfulness mean score	49	3.848 (0.834)	2.267–5.4
AQ-10 autism traits total score	49	2.408 (1.836)	0–8
**Candidate child predictors**			
Age (months)	52	35.483 (6.365)	17.54–44.02
ADOS-2 CSS autism symptoms	52	7.058 (1.697)	3–10
SCQ Autism symptoms total score	48	17.5 (5.527)	4–27
MSEL Developmental quotient	52	64.98 (26.054)	26.22– 135.266
VABS Adaptive behaviour SS	50	75.14 (10.864)	55–103
CBCL Emotional and behavioural difficulties			
Internalising	48	17.142 (8.276)	2–33.5
Externalising	48	19.618 (9.312)	4–41

*Note* One extreme outlier (for T1 VABS ABC) was Winsorised to within 2.5 standard deviations of the mean

*WEMWBS* Warwick-Edinburgh Mental Wellbeing Scale, *DASS-21* Depression Anxiety Stress Scales, *CQRS* Clarke Modification of the Holroyd Questionnaire on Resources and Stress, *MAAS* Mindful Attention Awareness Scale, *AQ-10* Autism Spectrum Quotient—10, *ADOS-2* Autism Diagnostic Observation Schedule—2nd Edition, *CSS* Calibrated Severity Scores, *SCQ* Social Communication Questionnaire, *MSEL* Mullen Scales of Early Learning, *VABS* Vineland Adaptive Behaviour Scales, *CBCL* Child Behaviour Checklist

### Predictors of Change in Wellbeing and Mental Health

Results from the regression models are presented in Table 5**.** All assumptions for regression analyses were met. In the prediction of WEMWBS T1-T2 RCI z-scores, the overall model was significant, with DASS-21 total score, CQRS family sharing, and VABS ABC SS entered as predictors together accounting for 22% of the variance. Among these, however, only VABS ABS SS was a significant unique predictor, accounting for 9% of the variance. In the model predicting T1-T3 WEMWBS RCI z-scores, the three candidate predictors (given n = 39) with strongest magnitude of effects were selected for inclusion. Together, DASS-21 total score, P-ESDM participation, and AFMSS initial statement accounted for 38% of the variance, with only P-ESDM participation making a significant unique contribution, accounting for 16% of variance (but noting a non-significant trend for contribution of DASS-21 score; *p* = 0.058).

**Table 5:** Standard Multiple Regressions for Predictors of Short-Term and Medium-Term Parental Wellbeing and Mental Health Problems

		Coefficients					Model summary			
		*B*	*SE B*	β	*t*	*p*	*R*^*2*^	*F*	*df*	*p*
*Wellbeing*										
T1-T2 WEMWBS RCI	DASS-21 total	0.013	0.011	0.179	1.190	0.241	0.222	3.712	3, 39	0.019
	CQRS family sharing	0.647	0.372	0.260	1.738	0.209				
	VABS ABC SS	0.037	0.018	0.298	2.102	0.042				
T1-T3 WEMWBS RCI	DASS-21 total	0.023	0.012	0.303	1.966	0.058	0.378	6.693	3, 33	0.001
	P-ESDM	1.139	0.381	0.415	2.986	0.005				
	AFMSS IS	0.742	0.421	0.275	1.765	0.087				
*Mental health*										
T1-T2 DASS-21 RCI	ADOS CSS	0.278	0.149	0.263	1.865	0.070	0.234	3.973	3, 39	0.015
	Brief COPE avoidant	−0.104	0.059	−0.288	−1.777	0.083				
	AFMSS IS	−0.764	0.581	−0.213	−1.315	0.196				
T1-T3 DASS-21 RCI	AFMSS IS	−1.567	0.585	−0.403	−2.680	0.011	0.403	7.184	1, 37	0.011

Unstandardised beta (*B*), standard error for unstandardized beta (*SE B*), standardised beta (β), t-score (*t*), p-value (*p*), R square (*R*^2^), F statistic (*F*), degrees of freedom (*df*)

*WEMWBS* Warwick-Edinburgh Mental Wellbeing Scale, *DASS-21* Depression Anxiety Stress Scales, *RCI* Reliable Change Index

For the model predicting T1-T2 DASS-21 RCI z-scores, 23% of variance was accounted for by ADOS CSS, Brief COPE avoidant coping, and AFMSS initial statement. However, no predictor made a significant unique contribution. For T1-T3 DASS-21 RCI scores, AFMSS initial statement was the only factor with a significant bivariate association, and entered into a regression model, accounted for 16% of the variance.

## Discussion

Many parents describe raising an Autistic child as a rewarding experience (Potter, 2016). However, arising challenges may substantially impact mental health and wellbeing (Schnabel et al., 2020). We found no mean-level group change in the mental health difficulties or wellbeing of parents of young Autistic pre-schoolers over a one-year period of accessing community-based early intervention services. However, substantial variability was evident with RCI z-scores for a small subgroup of parents indicative of ‘reliable’ change over our follow-up period.

### Predictors of Short-Term and Medium-Term Change in Wellbeing and Mental Health

Our hypothesis, that baseline parent-, child-, and family factors would predict change in parental mental health and wellbeing outcomes, was partially supported. After applying an FDR correction, the associations between baseline predictive factors and parent outcomes were no longer statistically significant; therefore, results from the regression analyses should be interpreted conservatively as trends given the increased chance of Type 1 error.

Results from regression analyses suggested that a combination of parent, child, and family/socioeconomic factors may predict change in parental wellbeing and mental health, consistent with Taraban and Shaw’s (2018) model of parenting. Parental coping strategies, specifically avoidant coping, were associated with short-term (T1-T2), but not medium-term (T1-T3), change in mental health challenges. These findings are consistent with past research demonstrating the importance of coping strategies to parental mental health and wellbeing (Benson, 2014; Cai et al., 2020; Dabrowska & Pisula, 2010; Zablotsky et al., 2013). Cai et al. (2020) found that avoidant coping strategies predicted anxiety and depression in mothers of young school-aged Autistic children, while problem-focused (approach) coping strategies predicted wellbeing. Cai et al.’s findings also highlight how mental health and wellbeing are related yet distinct constructs, with unique predictors. We also found that baseline measures of parent mental health challenges were associated with both short-term and medium-term change in wellbeing. This finding is consistent with past research demonstrating that concurrent measures of mental health predicted wellbeing, but not vice versa, among parents of Autistic pre-schoolers (Green et al., 2021).

Child factors were only associated with short-term, not medium-term, change in parent wellbeing and mental health outcomes. Parent ratings of children’s adaptive behaviour were associated with short-term change in parental wellbeing, while researcher ratings of child autism traits were associated with short-term change in parental mental health. Findings from past research examining the link between child characteristics and parent outcomes have been mixed. While adaptive behaviours have been associated with concurrent measures of parenting self-efficacy (Taylor et al., 2021) and sense of competence (Mathew et al., 2019), there has been little (Leadbitter et al., 2018) to no evidence (Green et al., 2021; Taylor et al., 2021) that adaptive behaviours have been directly linked to parent wellbeing. Two recent studies measured wellbeing using the WEMWBS in parents of Autistic children found no association between child adaptive behaviours and concurrent levels of parent wellbeing (Green et al., 2021; Taylor et al., 2021). It is therefore possible that while adaptive behaviour may not be associated with concurrent levels of wellbeing, it may be associated with change in wellbeing. Alternatively, methodological differences may account for conflicting findings between studies, as associations between adaptive behaviour and parent wellbeing have been found when using the recently-developed Autism Family Experience Questionnaire (AFEQ; Leadbitter et al., 2018). Some evidence suggests that adaptive behaviour may have been associated with concurrent parent mental health challenges in middle childhood (Salomone et al., 2018) but not during the pre-school years (Green et al., 2021). A study examining change in parenting stress in caregivers of Autistic pre-schoolers found that adaptive behaviour was predictive of change in parenting stress above and beyond cognitive skills, level of autism traits, and problem behaviour (Green & Carter, 2014). The current study examined changed in mental health challenges broadly, rather than its separate components (i.e., depression, anxiety, and stress); it is therefore possible that while we found no association between adaptive behaviour and mental health challenges, there may have been an association with parenting-related stress. Similarly, past findings regarding associations between child autism traits and parent outcomes have been mixed. Interestingly, researcher ratings of child autism traits have not been associated with concurrent measures of parent wellbeing or mental health (Green et al., 2021; Salomone et al., 2018); however, we have previously found that parent ratings of child autism traits were associated with concurrent levels of parent mental health, but not wellbeing, in a similar cohort (with some participants overlapping in the current sample) of parents of Autistic pre-schoolers (Green et al., 2021). The findings from the current study were therefore somewhat unexpected, in that researcher ratings, and not parent-ratings, of autism traits predicted short-term change in mental health. These findings draw attention to the differences that can occur due to the timing of measurements; however, caution should be taken in interpretation given the sample size.

The parent–child relationship, as measured by the AFMSS, was the only shared predictor of change in both wellbeing and mental health challenges. The AFMSS initial statement score reflects whether the first comment a parent makes about their child in response to a standard probe about their relationship is positive vs. neutral or negative (Benson et al., 2011). Here, this was predictive of variation in both short- and medium-term mental health change, and medium-term wellbeing change. That is, parents with subsequently positive shifts in mental health and wellbeing outcomes were more likely to have offered an initial positive or neutral (vs. negative) initial statement about their child, 5- to 10-months earlier. Research examining the predictive value of AFMSS ratings of parent–child relationship quality and parental mental health or wellbeing outcomes is limited, with sub-codes such as the Initial Statement rarely considered (Benson et al., 2011). The AFMSS was developed from the FMSS, which has evidence of robust association with mental health problems among parents of children with other conditions (Hastings et al., 2006). These findings signal the potential value of future research investigating current parent–child relationship quality as a potential indicator of future parent outcomes.

Among family/socioeconomic factors, only family support, as measured by the CQRS, was associated with short-term change in parental wellbeing. In families with Autistic children, family support networks have been shown to impact mental health (Bromley et al., 2004) and wellbeing (Ekas et al., 2010), and spousal support in particular has been shown to predict marital quality (Benson, 2020). Benson (2012) found that among mothers of Autistic children, the size and function (i.e., emotional support) of support networks predicted increased levels of perceived support, which predicted lower levels of depression and higher levels of wellbeing. Furthermore, received spousal support has been shown to indirectly effect marital quality, via perceived spousal support, only when child problem (externalising) behavious were at low or moderate levels, and not high (Benson, 2020). Benson’s (2020) findings suggest that spousal support may be most effective under less stressful parenting conditions. Together, these findings suggest the importance of support, particularly emotional support from family and significant others, for parents of Autistic children.

Finally, the potential impact of parent-mediated intervention on parental wellbeing and mental health was examined. Access to adjunctive parent-mediated intervention (between T2-T3) was associated with change in wellbeing outcomes immediately post-intervention (spanning T1-T3 follow-up). While several studies have reported benefits of parent-mediated intervention for parental wellbeing (Leadbitter et al., 2018; Palmer et al., 2020) and specific benefits of P-ESDM for stress (Estes et al., 2014) other studies have found no such effects (Green, 2024; Zhou et al., 2018). Mixed findings in the literature may be due to differences in study design, measurement, or analytic approach, or variation in participant/sample characteristics. Differential impacts for parent mental health and/or wellbeing may also be anticipated as a function of variation in the theoretical foundation, target outcomes, and/or delivery method of different parent-mediated interventions (Trembath et al., 2023).

Taken together, our findings highlight the potential influence that parent, child, and family factors may have on wellbeing and mental health in parents of Autistic children, in line with Taraban and Shaw’s (2018) adaptation of Belsky’s (1984) model of parenting. Furthermore, while our findings may be potentially contingent on specific test–retest intervals, they also highlight the importance of *timely* parent supports (e.g., coping strategies, social support networks), soon after diagnosis.

The primary limitation of this preliminary study is small sample size. Nevertheless, large effect sizes for observed associations (e.g., *d* = 0.79 for differential wellbeing outcomes from access to parent-mediated intervention group vs. rest of sample) suggests our reported results are robust. Furthermore, after correcting for multiple comparisons, initial associations between baseline predictor variables and parent outcomes were no longer significant, and hence results from the regression analyses should be considered with appropriate caution. Future larger studies are warranted to corroborate these, as well as potential genuine smaller effects that we might have been underpowered to identify, as well as test for potential moderating (e.g., parent–child relationship) and/or mediating (e.g., P-ESDM) effects. Exclusion criteria for the larger evaluation precluded the involvement of parents with significant unmanaged depression or anxiety, which may limit the generalisability of our findings. However, the factors included in our regression models all had moderate to strong bivariate associations with outcomes, so this exclusion on important ethical grounds unlikely substantially impacted our findings. The small number of fathers in the study impacts on the generalisability, and future studies may wish to consider targeted recruitment strategies to engage fathers.

Future investigation is also required to better understand the link between change in parental outcomes as a function of the parent–child relationship, indexed here by parent initial statement when describing their child. Perhaps most promising, our findings point toward potentially modifiable factors—engagement of coping strategies, access to informal social- and formal parent-mediated support services—that may be harnessed for the future benefit of parents of young Autistic children.

## Conclusion

Parents of Autistic pre-schoolers experienced lower levels of wellbeing and higher levels of mental health challenges compared to population norms in the period following their child’s diagnosis. However, a combination of parent, child, and family factors may predict change in wellbeing and mental health challenges. These findings are in line with Taraban and Shaw’s (2018) adaptation of Belsky’s (1984) model of parenting, suggesting that a complex interplay of factors impact on parental wellbeing and mental health. For clinicians working with families of Autistic children, our findings highlight the importance of initial parental mental health challenges, the quality of the parent–child relationship, and timely parental supports (e.g., developing coping strategies, support from family) for parents’ wellbeing and mental health following their child’s diagnosis.

## Competing Interests

Authors (CG, JS, CB, LC, KH) have previously received salary from grant funding to conduct research associated with the clinical provider of early intervention that participants were enrolled in, and thus held prior affiliations with this clinical provider.

## Supplementary Information

Below is the link to the electronic supplementary material.

Supplementary file1 (DOCX 26 KB)

Supplementary file2 (DOCX 33 KB)
