# Supplementary material for: Predictors of Change in Wellbeing and Mental Health of Parents of Autistic Pre-Schoolers
Source: J Autism Dev Disord. 2024 Jul 26;55(11):3798–810. doi: 10.1007/s10803-024-06471-7 (PMC12575482; doi:10.1007/s10803-024-06471-7)
Supplement: Supplementary file 1 — Supplementary file1 (DOCX 26 KB) [file 10803_2024_6471_MOESM1_ESM.docx]

| **Supplemental Table 1**  *Correlations between baseline characteristics and short-term and medium-term changes in parental wellbeing and mental health* | | | | | | | | | | | | | | | | | | |
| --- | --- | --- | --- | --- | --- | --- | --- | --- | --- | --- | --- | --- | --- | --- | --- | --- | --- | --- |
|  | | | | **Well-Being** | | | | | | |  | **Mental Health** | | | | | | |
|  | | | | **T1-T2**  **WEMWBS RCI** | | |  | **T1-T3**  **WEMWBS RCI** | | |  | **T1-T2**  **DASS-21 RCI** | | |  | **T1-T3**  **DASS-21 RCI** | | |
|  | | | | *n* | *r/r_s_* | *p* |  | *n* | *r/r_s_* | *p* |  | *n* | *r/r_s_* | *p* |  | *n* | *r/r_s_* | *p* |
| **T1 Parent Predictors** | | |  |  |  |  |  |  |  |  |  |  |  |  |  |  |  |  |
|  | Parent age (years) | |  | 44 | -0.025 | 0.0873 |  | 38 | 0.182 | 0.274 |  | 45 | 0.192 | 0.207 |  | 40 | -0.004 | 0.978 |
|  | DASS-21 Mental health difficulties total score | |  | 45 | 0.326 | 0.029 * |  | 38 | 0.420 | 0.009 * |  | - | - | - |  | - | - | - |
|  | WEMWBS Wellbeing total score | |  | - | - | - |  | - | - | - |  | 45 | 0.116 | 0.448 |  | 39 | -0.045 | 0.784 |
|  | CQRS Resources and stress | |  |  |  |  |  |  |  |  |  |  |  |  |  |  |  |  |
|  | | Family Sharing |  | 46 | 0.301 | 0.042 * |  | 39 | 0.359 | 0.025 * |  | 47 | 0.047 | 0.756 |  | 41 | 0.099 | 0.539 |
|  | | Supports |  | 46 | 0.13 | 0.387 |  | 39 | 0.102 | 0.536 |  | 47 | -0.155 | 0.299 |  | 41 | 0.086 | 0.591 |
|  | Brief COPE Coping mechanisms | |  |  |  |  |  |  |  |  |  |  |  |  |  |  |  |  |
|  | | Avoidant |  | 46 | 0.164 | 0.276 |  | 39 | 0.399 | 0.012 * |  | 45 | -0.377 | 0.011 * |  | 39 | -0.0311 | 0.054 |
|  | | Approach |  | 46 | -0.14 | 0.353 |  | 39 | -0.207 | 0.206 |  | 45 | -0.097 | 0.528 |  | 39 | 0.12 | 0.468 |
|  | MAAS Mindfulness mean score | |  | 46 | 0.039 | 0.797 |  | 39 | -0.084 | 0.613 |  | 45 | -0.143 | 0.348 |  | 39 | -0.269 | 0.098 |
|  | AQ-10 autism traits total score | |  | 46 | 0.13 | 0.389 |  | 39 | 0.304 | 0.06 |  | 45 | -0.112 | 0.465 |  | 39 | -0.049 | 0.766 |
|  | Autism-Specific Five Minute Speech Sample | |  |  |  |  |  |  |  |  |  |  |  |  |  |  |  |  |
|  |  | Critical comments |  | 45 | -0.035 | 0.819 |  | 38 | -0.08 | 0.634 |  | 46 | 0.132 | 0.381 |  | 40 | -0.002 | 0.99 |
|  |  | Positive comments |  | 45 | 0.029 | 0.848 |  | 38 | -0.039 | 0.814 |  | 46 | 0.034 | 0.825 |  | 40 | 0.175 | 0.281 |
| **T1 Child Predictors** | | |  |  |  |  |  |  |  |  |  |  |  |  |  |  |  |  |
|  | Age (months) | |  | 46 | 0.050 | 0.740 |  | 39 | 0.125 | 0.449 |  | 47 | 0.093 | 0.536 |  | 41 | 0.051 | 0.749 |
|  | ADOS-2 CSS Autism Symptoms | |  | 46 | -0.119 | 0.430 |  | 39 | -0.362 | 0.024 * |  | 47 | 0.291 | 0.047 * |  | 41 | 0.117 | 0.464 |
|  | SCQ Autism Symptoms total score | |  | 43 | -0.071 | 0.653 |  | 36 | -0.180 | 0.294 |  | 44 | -0.034 | 0.827 |  | 38 | -0.113 | 0.500 |
|  | MSEL Developmental Quotient | |  | 46 | 0.252 | 0.091 |  | 39 | 0.346 | 0.031 * |  | 47 | -0.073 | 0.625 |  | 41 | 0.091 | 0.570 |
|  | VABS Adaptive Behaviour SS | |  | 44 | 0.299 | 0.049 * |  | 37 | 0.196 | 0.245 |  | 45 | -0.110 | 0.471 |  | 39 | 0.042 | 0.799 |
|  | CBCL Emotional and Behavioural difficulties | |  |  |  |  |  |  |  |  |  |  |  |  |  |  |  |  |
|  | | Internalising |  | 44 | 0.050 | 0.745 |  | 37 | -0.042 | 0.806 |  | 45 | 0.042 | 0.785 |  | 38 | -0.041 | 0.805 |
|  | | Externalising |  | 44 | 0.139 | 0.369 |  | 37 | -0.033 | 0.848 |  | 45 | 0.041 | 0.788 |  | 38 | 0.016 | 0.998 |
| Positive WEMWBS RCI scores indicate an increase in well-being. Positive DASS-21 RCI scores indicate an increase mental health problems.  * *p* < 0.05 without correction for multiple comparisons. After applying the Benjamini-Hochberg (False Discovery Rate) correction, findings were no longer significant. This correction may be too stringent, and given that the sizes of the correlation coefficients indicate small to medium effects (Ferguson, 2016), results are reported without correction for multiple comparisons.  WEMWBS: Warwick-Edinburgh Mental Wellbeing Scale; DASS-21: Depression Anxiety Stress Scales; RCI: Reliable Change Index; CQRS: Clarke modification of the Holroyd Questionnaire on Resources and Stress; MAAS: Mindful Attention Awareness Scale; AQ-10: Autism Spectrum Quotient – 10; ADOS-2: Autism Diagnostic Observation Schedule – 2^nd^ edition. CSS: Calibrated Severity Scores; SCQ: Social Communication Questionnaire; MSEL: Mullen Scales of Early Learning; VABS: Vineland Adaptive Behaviour Scales; CBCL: Child Behaviour Checklist. | | | | | | | | | | | | | | | | | | |
