# Supplementary material for: Predictors of Change in Wellbeing and Mental Health of Parents of Autistic Pre-Schoolers
Source: J Autism Dev Disord. 2024 Jul 26;55(11):3798–810. doi: 10.1007/s10803-024-06471-7 (PMC12575482; doi:10.1007/s10803-024-06471-7)
Supplement: Supplementary file 2 — Supplementary file2 (DOCX 33 KB) [file 10803_2024_6471_MOESM2_ESM.docx]

| **Supplemental Table 2** | | | | | | | | | | | | | | | | | | | | | |
| --- | --- | --- | --- | --- | --- | --- | --- | --- | --- | --- | --- | --- | --- | --- | --- | --- | --- | --- | --- | --- | --- |
| *Differences in Candidate Predictors for Parent Outcomes* | | | | | | | | | | | | | | | | | | | | | |
|  | |  | **Wellbeing** | | | | | | | | |  | **Mental Health** | | | | | | | | |
|  | |  | **T1-T2**  **WEMWBS RCI** | | | |  | **T1-T3**  **WEMWBS RCI** | | | |  | **T1-T2**  **DASS-21 RCI** | | | |  | **T1-T3**  **DASS-21 RCI** | | | |
|  | |  | *M* (*SD*) | *t/F (df)* | *p* | d/η² |  | *M* (*SD*) | *t/F (df)* | *p* | d/η² |  | *M* (*SD*) | *t/F (df)* | *p* | d/η² |  | *M* (*SD*) | *t/F (df)* | *p* | d/η² |
| **Parent** | | | | | | | | | | | | | | | | | | | | | |
|  | Mother |  | -0.16(1.47) | 0.58(44) | 0.565 | -0.27 |  | -0.32(1.38) | -0.06(37) | 0.950 | -0.03 |  | 0.22(1.19) | 0.64(45) | 0.524 | 0.30 |  | 0.52(2.00) | 1.23(39) | 0.226 | 0.59 |
|  | Father |  | 0.23(0.75) |  |  |  |  | -0.28(1.09) |  |  |  |  | -0.32(0.68) |  |  |  |  | -0.61(1.12) |  |  |  |
|  |  |  |  |  |  |  |  |  |  |  |  |  |  |  |  |  |  |  |  |  |  |
| **Parent-Mediated Intervention** | | | | | | | | | | | | | | | | | | | | | |
|  | P-ESDM |  | - | - | - | - |  | 0.30 (1.19) | -2.40(37) | 0.022* | -0.79 |  | - | - | - | - |  | 0.17(1.34) | 0.55(39) | 0.586 | 0.18 |
|  | No P-ESDM |  | - |  |  |  |  | -0.70 (1.29) |  |  |  |  | - |  |  |  |  | 0.52(2.26) |  |  |  |
|  |  |  |  |  |  |  |  |  |  |  |  |  |  |  |  |  |  |  |  |  |  |
| **Parent Education** | | | | | | | | | | | | | | | | | | | | | |
|  | ≤ Secondary |  | -1.02(1.55) | -1.50(40) | 0.141 | -0.66 |  | -0.47(2.04) | -0.31(33) | 0.759 | -0.15 |  | -0.48(1.99) | -1.03(41) | 0.311 | -0.42 |  | 0.72(2.02) | 0.43(35) | 0.670 | 0.19 |
|  | Tertiary |  | -0.07(1.40) |  |  |  |  | -0.25(1.31) |  |  |  |  | 0.26(1.72) |  |  |  |  | 0.34(1.99) |  |  |  |
|  |  |  |  |  |  |  |  |  |  |  |  |  |  |  |  |  |  |  |  |  |  |
| **Parent Ethnicity** | | | | | | | | | | | | | | | | | | | | | |
|  | Australian |  | -0.04(1.21) | 0.40(43) | 0.692 | 0.12 |  | -0.16(1.10) | 0.60(36) | 0.553 | 0.20 |  | -0.08(1.58) | -0.68(44) | 0.498 | -0.21 |  | 0.39(2.24) | -0.01(38) | 0.992 | -0.00 |
|  | Other |  | -0.21(1.55) |  |  |  |  | -0.42(1.52) |  |  |  |  | 0.30(1.92) |  |  |  |  | 0.39(1.83) |  |  |  |
|  |  |  |  |  |  |  |  |  |  |  |  |  |  |  |  |  |  |  |  |  |  |
| **Autism-Specific Five-Minute Speech Sample** | | | | | | | | | | | | | | | | | | | | | |
|  | **Initial Statement** | | | | | | | | | | | | | | | | | | | | |
|  | Neutral |  | -.39(1.45) | -1.22(42) | 0.229 | -.37 |  | -0.77(1.50) | -2.185(36) | 0.035* | 0.709 |  | 0.71(1.49) | 2.22(43) | 0.031* | 0.656 |  | 1.14(1.826) | 2.68(37) | 0.011* | 0.861 |
|  | Positive |  | 0.13(1.3) |  |  |  |  | 0.14(1.02) |  |  |  |  | -0.44(1.99) |  |  |  |  | -0.43(1.813) |  |  |  |
|  | **Warmth** |  |  |  |  |  |  |  |  |  |  |  |  |  |  |  |  |  |  |  |  |
|  | High |  | 0.38 (1.36) | 1.58(2,42) | 0.218 | 0.070 |  | 0.35 (1.16) | 2.469(2,35) | 0.099 | 1.124 |  | 0.070 (2.24) | 0.08(2,43) | 0.921 | 0.004 |  | 0.43 (2.09) | 0.01(2,37) | 0.990 | 0.000 |
|  | Moderate |  | -0.14 (0.21) |  |  |  |  | -0.37 (1.62) |  |  |  |  | 0.00 (0.81) |  |  |  |  | 0.36 (1.29) |  |  |  |
|  | Low |  | -0.43(1.51) |  |  |  |  | -0.69 (1.30) |  |  |  |  | 0.27 (1.70) |  |  |  |  | 0.33 (2.09) |  |  |  |
|  | **Relationship** |  |  |  |  |  |  |  |  |  |  |  |  |  |  |  |  |  |  |  |  |
|  | Negative /Neutral |  | -0.07(1.36) | 0.49(43) | 0.625 | 0.156 |  | -0.39(1.45) | -0.514(36) | 0.610 | -0.179 |  | 0.10(1.750) | -0.40(44) | 0.688 | -0.127 |  | -0.39 (1.45) | -0.51(36) | 0.610 | -0.179 |
|  | Positive |  | -0.29 (1.54) |  |  |  |  | -0.15 (1.15) |  |  |  |  | 0.33 (1.92) |  |  |  |  | -0.15 (1.15) |  |  |  |
|  |  |  |  |  |  |  |  |  |  |  |  |  |  |  |  |  |  |  |  |  |  |
| **Household Income** | | | | | | | | | | | | | | | | | | | | | |
|  | Low-income |  | 0.39(1.07) | -1.99(42) | 0.053 | -0.63 |  | 0.08(0.65) | -1.32(35) | 0.194 | -0.47 |  | -0.37(1.41) | 1.62(42) | 0.112 | 0.52 |  | -0.15(1.85) | 1.61(37) | 0.117 | 0.55 |
|  | Not low income |  | -0.48(1.52) |  |  |  |  | -0.54(1.56) |  |  |  |  | 0.52(1.86) |  |  |  |  | 0.84(1.78) |  |  |  |
|  |  |  |  |  |  |  |  |  |  |  |  |  |  |  |  |  |  |  |  |  |  |
| **Primary Language** | | | | | | | | | | | | | | | | | | | | | |
|  | English |  | -0.19(1.34) | -0.35(43) | 0.728 | -0.12 |  | -0.47(1.36) | -1.16(36) | 0.254 | -0.42 |  | 0.28(1.54) | 0.72(44) | 0.46 | 0.23 |  | 0.53(1.83) | 0.62(38) | 0.540 | 0.21 |
|  | Other |  | -0.02(1.64) |  |  |  |  | 0.09(1.31) |  |  |  |  | -0.18(2.41) |  |  |  |  | 0.11(2.27) |  |  |  |
|  |  |  |  |  |  |  |  |  |  |  |  |  |  |  |  |  |  |  |  |  |  |
| **Number of Children** | | | | | | | | | | | | | | | | | | | | | |
|  | 1 child |  | 0.23(1.39) | 1.34(43) | 0.188 | 0.42 |  | 0.10(1.23) | 1.50(36) | 0.154 | 0.49 |  | 0.27(2.00) | 0.33(44) | 0.746 | 0.100 |  | 0.49(2.05) | 0.27(38) | 0.791 | 0.09 |
|  | > 1 child |  | -0.35(1.40) |  |  |  |  | -0.55(1.38) |  |  |  |  | 0.10(1.69) |  |  |  |  | 0.32(1.95) |  |  |  |
|  |  |  |  |  |  |  |  |  |  |  |  |  |  |  |  |  |  |  |  |  |  |
| **No. Children with Autism** | | | | | | | | | | | | | | | | | | | | | |
|  | Simplex family |  | -0.21(1.48) | -0.72(43) | 0.473 | -0.30 |  | -0.37(1.40) | -0.76(36) | 0.454 | -0.40 |  | 0.19(1.91) | 0.29(44) | 0.771 | 0.12 |  | 0.53(2.03) | 1.19(38) | 0.242 | 0.57 |
|  | Multiplex family |  | 0.21(0.96) |  |  |  |  | 0.18(0.75) |  |  |  |  | -0.02(1.02) |  |  |  |  | -0.58(1.11) |  |  |  |
|  |  |  |  |  |  |  |  |  |  |  |  |  |  |  |  |  |  |  |  |  |  |
| **Assessed during COVID-19 Pandemic** | | | | | | | | | | | | | | | | | | | | | |
|  | Before |  | -0.26(1.47) | -1.33(44) | -0.19 | -0.48 |  | -0.36(1.51) | -0.36(37) | 0.720 | -0.13 |  | 0.28(1.92) | 0.87(45) | 0.387 | 0.31 |  | 0.69(1.88) | 1.71(39) | 0.095 | 0.602 |
|  | During |  | 0.41(1.09) |  |  |  |  | -0.19(0.74) |  |  |  |  | -0.27(0.99) |  |  |  |  | -0.46(1.96) |  |  |  |
| Positive WEMWBS RCI scores indicate an increase in well-being. Positive DASS-21 RCI scores indicate an increase mental health problems.  * *p <* 0.05 without correction for multiple comparisons. After applying the Benjamini-Hochberg (False Discovery Rate) correction, findings were no longer significant. This correction may be too stringent, and given that the effect sizes indicate small to medium effects (Ferguson, 2016), results are reported without correction for multiple comparisons.  WEMWBS: Warwick-Edinburgh Mental Wellbeing Scale; DASS-21: Depression Anxiety Stress Scales; RCI: Reliable Change Index; P-ESDM: Parent-Mediated Early Start Denver Model | | | | | | | | | | | | | | | | | | | | | |
